# Flavones, Flavonols, and Glycosylated Derivatives—Impact on *Candida albicans* Growth and Virulence, Expression of *CDR1* and *ERG11*, Cytotoxicity

**DOI:** 10.3390/ph14010027

**Published:** 2020-12-30

**Authors:** Marija Ivanov, Abhilash Kannan, Dejan S. Stojković, Jasmina Glamočlija, Ricardo C. Calhelha, Isabel C. F. R. Ferreira, Dominique Sanglard, Marina Soković

**Affiliations:** 1 Department of Plant Physiology, Institute for Biological Research “Siniša Stanković”, National Institute of Republic of Serbia, University of Belgrade, Bulevar Despota Stefana 142, 11000 Belgrade, Serbia; marija.smiljkovic@ibiss.bg.ac.rs (M.I.); dejanbio@ibiss.bg.ac.rs (D.S.S.); jasna@ibiss.bg.ac.rs (J.G.); 2 Institute of Microbiology, University Hospital Lausanne and University Hospital Center, Rue du Bugnon 48, 1011 Lausanne, Switzerland; abhilifescizurich@gmail.com (A.K.); dominique.sanglard@chuv.ch (D.S.); 3 Centro de Investigação de Montanha (CIMO), Instituto Politécnico de Bragança, Campus de Santa Apolónia, 5300-253 Bragança, Portugal; calhelha@ipb.pt (R.C.C.); iferreira@ipb.pt (I.C.F.R.F.)

**Keywords:** flavonoids, isoquercitrin, antifungal, biofilm, antivirulence, efflux pumps, cytotoxicity

## Abstract

Due to the high incidence of fungal infections worldwide, there is an increasing demand for the development of novel therapeutic approaches. A wide range of natural products has been extensively studied, with considerable focus on flavonoids. The antifungal capacity of selected flavones (luteolin, apigenin), flavonols (quercetin), and their glycosylated derivatives (quercitrin, isoquercitrin, rutin, and apigetrin) along with their impact on genes encoding efflux pumps (*CDR1*) and ergosterol biosynthesis enzyme (*ERG11*) has been the subject of this study. Cytotoxicity of flavonoids towards primary liver cells has also been addressed. Luteolin, quercitrin, isoquercitrin, and rutin inhibited growth of *Candida albicans* with the minimal inhibitory concentration of 37.5 µg/mL. The application of isoquercitrin has reduced *C. albicans* biofilm establishing capacities for 76%, and hyphal formation by yeast. In vitro treatment with apigenin, apigetrin, and quercitrin has downregulated *CDR1*. Contrary to rutin and apigenin, isoquercitrin has upregulated *ERG11*. Except apigetrin and quercitrin (90 µg/mL and 73 µg/mL, respectively inhibited 50% of the net cell growth), the examined flavonoids did not exhibit cytotoxicity. The reduction of both fungal virulence and expression of antifungal resistance-linked genes was the most pronounced for apigenin and apigetrin; these results indicate flavonoids’ indispensable capacity for further development as part of an anticandidal therapy or prevention strategy.

## 1. Introduction

*Candida albicans* is nowadays recognized as one of the most common human pathogens. Its significance as a serious health as well as economic burden is a consequence of high mortality rates (>70%) in some groups of patients, mainly immunocompromised [1]. Yeast *C. albicans* is the predominant cause of vulvovaginal candidiasis, a disease affecting up to 75% of women once in a lifetime [2], as well as oral candidiasis [3] and the prevalent healthcare-associated invasive fungal infection—invasive candidiasis [4]. Besides posing a serious threat to human health it is also the causative agent of animal skin infections [5].

This opportunistic fungus has developed a wide network of adaptations in order to survive and spread throughout human organism. It can group into biofilms, transit between yeast and hyphal morphology, and produce hydrolytic enzymes [6]. On the other hand, *C. albicans* also uses certain adaptation traits, including increased expression of *CDR1* and *ERG11* genes, to avoid elimination by antifungal therapeutics [7]. All of the abovementioned traits have contributed to the medical significance of this pathogen—*C. albicans* is worldwide listed as one of the most common causes of human fungal infections with around 700,000 cases of invasive and 2,000,000 cases of oral candidiasis recorded annually [8]. The extensive usage of current first line antifungals is being linked to different side effects, including hepatotoxicity and nephrotoxicity, along with increased frequency of strains resistant to this type of treatment [9], urging the development of novel therapies.

Natural products have been extensively studied—at first in order to find agents that could inhibit growth of pathogenic microorganisms, but recently attention is focused on the role of natural products as inhibitors of virulence and quorum quenching agents [10]. Biological activities of numerous medicinal plants and foods are linked to abundant presence of flavonoids as bioactive principles. Besides well-known antioxidant properties of these molecules, there have been also studies regarding their antifungal potential. However, despite numerous researches in this field, mechanisms of antifungal activities as well as antivirulence properties of flavonoids remain mainly unrevealed [11].

The following study addressed anti-candidal activity of selected flavonoids: Flavone aglycones (luteolin, apigenin), a flavone glycosylated derivative (apigetrin), flavonol (quercetin), and its glycosylated derivatives (quercitrin, isoquercitrin, rutin). These molecules are present ubiquitously within plant kingdom and this study aimed to provide an insight into their antifungal potential with special focus on antivirulence properties along with impact on resistance linked genes expression as promising novel antifungal strategies.

## 2. Results

### 2.1. Antifungal Potential of Selected Flavonoids

The best antifungal potential among the tested compounds could be observed for luteolin, quercitrin, isoquercitrin, and rutin (minimum inhibitory concentration (MIC) 37.5 µg/mL) (Table 1). Apigenin and apigetrin were the subject of a previous study by our group [12], and additional insight into their anticandidal activities has been enlightened within this study. Previously published active concentrations of apigenin (MIC 100 µg/mL) and apigetrin (MIC 50 µg/mL) towards the strain *C. albicans* 475/15 [12] were used in further experiments.

### 2.2. Interference of Tested Flavonoids with C. albicans Biofilm Formation

**Figure 1:**
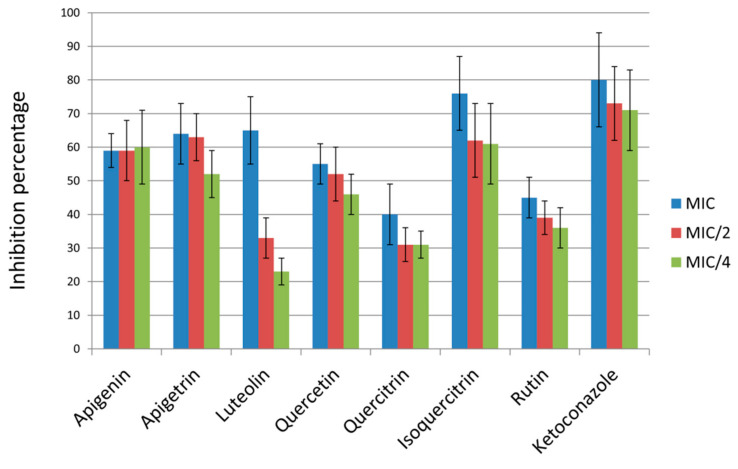
Percentage of inhibition of C. *albicans* biofilm formation after treatment with MIC and subMICs (MIC/2 and MIC/4) of compounds. Values are means ± SD of three replicates.

All of the tested compounds exhibited some ability to act as biofilm formation inhibitors, especially when applied in concentrations equal to MIC or MIC/2. The most promising effect at MIC concentrations could be noticed for isoquercitrin (76% inhibition) while both apigetrin and isoquercitrin applied in MIC/2 were able to prevent biofilm formation > 60%. On the other hand, application of quercitrin and rutin MIC reduced formation of biofilms for less than 50% (Figure 1).

### 2.3. Flavonoids as Inhibitors of C. albicans Hyphal Growth

**Figure 2:**
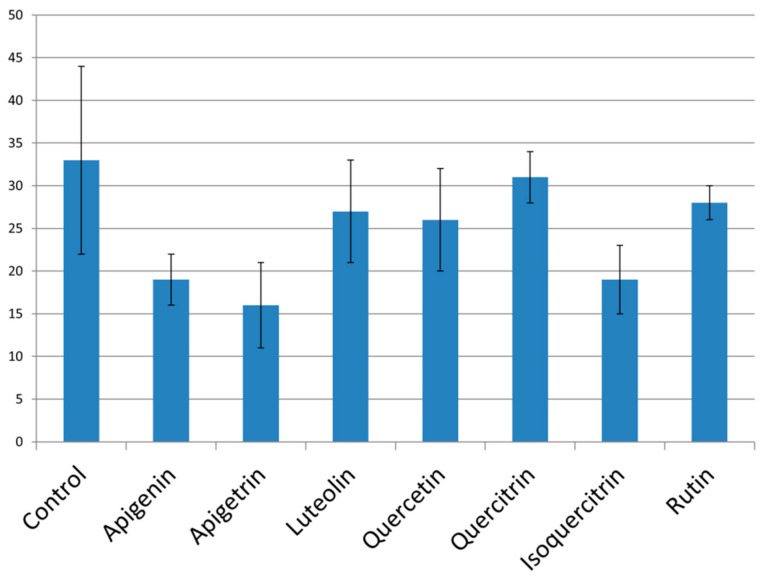
Percentage of hyphal cells calculated by comparison with the total number of *C. albicans* cells. Values are means ± SD of three replicates.

The examined flavonoids have mainly shown moderate activity in the terms of reducing fungal hyphal growth (Figure 2). Treatment of *C. albicans* cells with both apigetrin and its aglycone apigenin has led to the lowest number of cells growing in the hyphal form (Figure 2). These two flavones have, in a previous experiment, reduced biofilm formation for more than 50% implying their overall great potential for reduction of fungal virulence capacity. Isoquercitrin has also been efficient in reduction of hyphal growth, and since it had lower MIC values than apigenin and apigetrin [12], this activity has been accomplished in the lowest concentration among the tested molecules.

### 2.4. Expression of Antifungal Resistance Associated Genes after Application of Flavonoids

**Figure 3:**
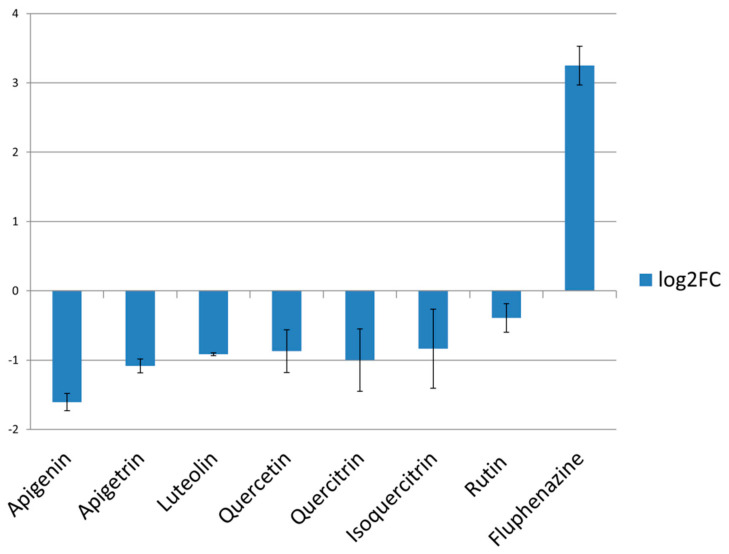
Expression levels *CDR1* after treatment of MIC of compounds. Fluphenazine was used as positive control. Values are expressed as Log2 fold changes (log2FC) of Relative Quantification (RQ) values, averages of two biological replicates.

Treatment of *C. albicans* cells with selected flavonoids led to a more-or-less significant reduction of *CDR1* expression levels. Apigenin and apigetrin exhibited the most prominent impact on lowering *CDR1* levels (log2FC < −1), while the effect of other flavonoids was less profound (Figure 3).

**Figure 4:**
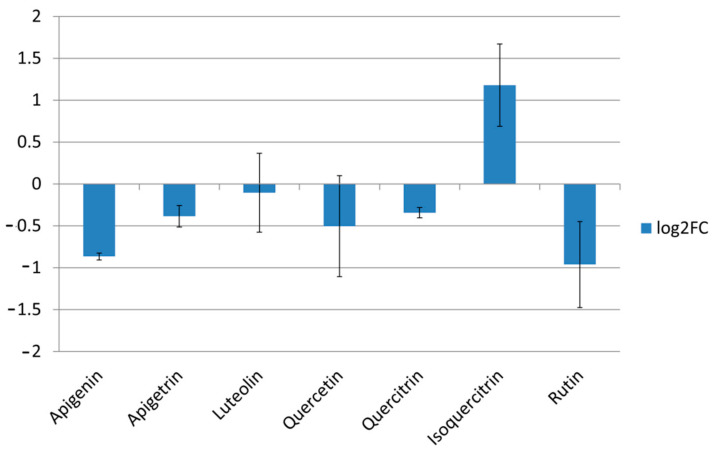
Expression levels of *ERG11* after treatment of MIC of compounds. Values are expressed as Log2 fold changes of RQ values, averages of two biological replicates.

Treatment with isoquercitrin has up-regulated expression of *ERG11* in *C. albicans* cells (Log2FC > 1), an undesirable trait of potential antifungals since their application can lead to reduced susceptibility to azole antifungals. Treatment of *C. albicans* with apigenin and rutin led to lower expression levels of *ERG11* (Figure 4), suggesting that these compounds have potential for further development as part of novel antifungal strategies.

### 2.5. Cytotoxicity of Selected Compounds

Apigenin, luteolin, quercetin, isoquercitrin, and rutin did not exhibit cytotoxic effect towards porcine liver cells PLP2 (Table 2). On the other hand, apigetrin and quercitrin induced cytotoxicity at GI_50_ 90 µg/mL and 73 µg/mL, respectively.

## 3. Discussion

Previous studies [13,14,15] have suggested less promising antifungal activity of luteolin towards different *C. albicans* strains (MIC 3.1 mg/mL, MIC > 83 µg/mL, and >1 mg/mL, respectively) compared to 37.5 µg/mL in this study (Table 1). Although quercetin in our study exhibited MIC 75 µg/mL (Table 1), much better activity has been detected previously [16] (MIC 8 µg/mL towards *C. albicans* ATCC 10231). On the other hand, lower activity of quercetin was observed in previous studies: MIC 100 µg/mL, *C. albicans* ATCC 10231 [17], MIC > 83 µg/mL, clinical isolates, and *C. albicans* 10231 [14], and MIC 128–512 µg/mL, clinical isolates, and reference *C. albicans* strains ATCC 10231 and SC5314 [18]. The obtained information regarding excellent antifungal potential of the flavonol glycoside, quercitrin (MIC 37.5 µg/mL) (Table 1), differs from studies by Gehrke et al. [17] and Sekita et al. [19] where MICs 100 µg/mL (towards *C. albicans* ATCC 10231) and >512 µg/mL (*C. albicans* CAD1, clinical isolate), respectively, were found. Isoquercitrin in our study exhibited MIC 37.5 µg/mL (Table 1) while an earlier study [20] established much better antifungal potency with MIC 2.5 µg/mL towards *C. albicans* ATCC 90028. Rutin had excellent activity in inhibiting growth of *C. albicans* (MIC 37.5 µg/mL, Table 1), while similar antimicrobial potential of this flavonoid has been determined previously [21], (MIC 40 µg/mL towards *C. albicans* ATCC 10231). Lower antimicrobial potential of rutin has been established in the studies by Han et al. [22] where 1000 µg/mL of rutin was necessary for *C. albicans* CA-1 growth inhibition and in the studies by Johann et al. [23] where it was claimed that rutin has insignificant antifungal potential (MIC > 1000 µg/mL) when applied towards *C. albicans* ATCC 18804. There could be various reasons that led to manifested differences in antimicrobial potential of all the mentioned compounds. Among the many of potential reasons we could highlight differences in strains sensitivity since different strains of *C. albicans* were used in almost all referred studies. We could observe stronger antifungal activity of glycosylated flavonol derivatives (quercitrin, isoquercitrin, and rutin) compared to their aglycone, quercetin (MIC 37.5 µg/mL and 75 µg/mL, respectively). Sugar moiety has also been highlighted as the reason for previously confirmed stronger antifungal effect of apigenin-7*O*-glycoside compared to its aglycone apigenin [12]. This superiority might be related to increased solubility and amphiphilicity recorded for glycosylated plant metabolites, which may enhance their membrane transport [24].

Apigenin in the previous study [25] did not show any antibiofilm effect when concentration 125 µg/mL was applied towards oral and reference *C. albicans* isolates (SC5314 and 3153A). On the other hand, another study [26] determined its high efficiency since only 5 µg/mL of this flavonoid was necessary for *C. albicans* ATCC 90028 biofilm reduction. Luteolin investigation [27] claimed that this flavonoid is inefficient towards *C. albicans* ATCC 90028 biofilm in much higher concentrations (625–5000 µg/mL) than examined in our study (9.38–37.5 µg/mL). The impact of quercetin on *C. albicans* NBC099 biofilms was studied earlier [28] but in this investigation, quercetin was applied in a much higher concentration that prevented biofilm establishment (200 μg/mL compared to 18.75–75 µg/mL in our study). Other studies have examined impact of this flavonol on preformed *C. albicans* ATCC 90028 biofilms—a concentration of 625 µg/mL was inactive against 24-h-old biofilms [27]. Lower antibiofilm activity of quercetin has been observed by determination of SMIC (sessile cells MIC); SMIC > 200 µg/mL (oral and reference *C. albicans* isolates (SC5314 and 3153A, [25]) and SMIC ≥ 1024 µg/mL (*C. albicans* ATCC 10231, SC 5314, and clinical isolates, [18]). Cells in the established biofilms are more resistant to antifungal treatment [29] so this might be the reasons for determined high SMICs. Further insight into antibiofilm potential of quercetin has been provided in the study by Gao et al. [18] where it was observed that treatment with this flavonoid leads to reduced *ALS1*, *ALS3* (Agglutinin Like Sequence gene family), *HWP1* (Hyphal Wall Protein), and *SUN41* (gene encoding for secreted beta-glucosidase) expression levels. Quercitrin had the lowest potential to reduce biofilm formation among all the flavonoids tested in our study (Figure 1) with its complete lack of activity towards *C. albicans* CAD1 biofilm observed previously [19]. On the other hand, excellent activity on inhibition of *C. albicans* biofilm formation was addressed to another flavonol glycoside, isoquercitrin (tested concentration 9.38–37.5 µg/mL) (Figure 1) with a previous study [19] highlighting its antibiofilm potential in higher concentration—200 μg/mL. Rutin was among the natural compounds with the lowest activity against biofilm formation in this study (Figure 1) with its lack of *C. albicans* CAD1 antibiofilm activity also observed earlier [19]. Overall, glycoside derivatives, apigetrin and isoquercitrin, have shown more promising antibiofilm potential compared to their corresponding aglycones. The superior antibiofilm potential of isoquercitrin compared to the other quercetin glycosides examined (quercitrin and rutin) suggests that biological activity is strongly enhanced by beta-d-glucosyl residue compared to alpha-l-rhamnosyl moiety (quercitrin) and 3-*O*-rutinoside (rutin). A recent study [30] indicated rutin as the least active quercetin derivative, which is by some extent confirmed in the antibiofilm and antihyphal assays in our study, especially in comparison with isoquercitrin. The less promising antibiofilm potential of quercitrin and rutin, compared to isoquercitrin, might be linked to their higher lipophilicity since the increased lipophilicity might reduce membrane-passing capability [30].

The strongest anti-hyphal potential was observed for apigetrin, a glycoside not studied before as inhibitor of this virulence trait, according to the author’s best knowledge. On the other hand, the potential of quercetin to reduce formation of hyphae has been studied previously. Application of up to 200 µg/mL [28] and 64 µg/mL [18] of quercetin led to reduction of morphological transition of this yeast, while in our study, 75 µg/mL has induced only slight inhibition.

The inhibitory impact of two flavones, apigenin and apigetrin (Figure 3), along with previously published flavonol astragalin [31] on *CDR1* expression should be further explored since their application might increase fungal susceptibility to current therapeutics due to reduction in cell efflux. Apigenin has been examined previously when applied 38 µg/mL inhibited bacterial efflux pumps [32]. Application of quercetin and luteolin caused *S. aureus* efflux inhibition with IC_50_ 75 µg/mL [32], while in our study, they did not exhibit any significant inhibitory potential towards *CDR1* expression when applied in 75 µg/mL and 37.5 µg/mL, respectively. Isoquercitrin treatment has increased expression levels of *ERG11* (Figure 4) proposing potential negative interaction of this flavonoid with azole antifungals targeting enzyme CYP51 encoded by *ERG11*. Apigenin and rutin, due to their negative impact on *ERG11* expression (Figure 4), might help in eradicating the infections caused by *C. albicans* strains resistant to azole drugs due to mutations that upregulate *ERG11*. These presumptions should be further investigated. As far as we know, none of these compounds have been tested earlier in order to examine their impact on *ERG11* and *CDR1* expression; furthermore, their downregulating ability is of great interest especially bearing in mind the lack of ketoconazole, a widely used antifungal, and the impact on downregulation of *CDR1* and *ERG11* determined in the identical assay [31].

Previous study of apigenin claimed that it induces cytotoxicity mainly in the cancer cell lines [33]. Apigenin has exhibited cytotoxicity towards TIG-1 (human lung embryonic fibroblasts) and HUVE (human umbilical vein endothelial) cells (LC_50_ 110 µM) [34] with LC_50_ value lower than apigenin MIC (Table 1) suggesting that despite its non-cytotoxicity towards PLP2 cells (Table 2) its cytotoxicity towards additional cell lines should be further explored. Glycoside of apigenin and apigetrin, showed some cytotoxic effect (Table 2) in concentrations higher than its average MICs [12]; meanwhile, previous study [35] demonstrated that apigetrin concentration up to 100 μM does not induce cytotoxicity in 3T3-L1 pre-adipocytes. Luteolin in previous studies showed LC_50_ 107 μM and 57 μM towards TIG-1 and HUVE cells, respectively [34]. The study of luteolin effect on apoptosis of normal human peripheral blood mononuclear cells showed that it does not induce apoptosis in concentration tested (20 μM) [36]. Quercetin did not exhibit cytotoxicity towards liver cells in our study, unlike in another study [34] where cytotoxicity towards HUVE cells was determined (LC_50_ 61 µM). Quercetin glycoside, quercitrin, exhibited GI_50_ 73 µg/mL (Table 2), which is lower than its MFCs (Table 1) implying that side effects may arise as a consequence of quercitrin antifungal application. Another quercetin glycoside, isoquercitrin, did not show cytotoxic effects towards tested cells in this study (Table 2), as well as towards non-tumor colon cell (IEC-18) (up to 150 μM concentration) [37]. Rutin did not show cytotoxic effect in our study (Table 2), which is in line with previous study on different cells [34].

## 4. Materials and Methods

### 4.1. Fungal Culture Conditions

*C. albicans* was isolated from oral cavities of patients at ENT Clinic, Clinical Hospital Centre Zvezdara, Belgrade, Serbia after obtaining informed written consent and identified by using CHROMagar plates (Biomerieux, Craponne, France). Strains were maintained on Sabouraud Dextrose Agar (Merck, Darmstadt, Germany) and are deposited at the Mycological Laboratory, Department of Plant Physiology, Institute for Biological Research “Siniša Stanković”, National Institute of Republic of Serbia, University of Belgrade. The collection of samples was approved by the Ethical Committee (Office for Human Research Protection, Zvezdara University Medical Center, Belgrade, Serbia, document issued 26 October 2016).

### 4.2. Antifungal Activity

Microdilution assay [38] with some modification was used for determination of minimum inhibitory (MIC) and minimum fungicidal concentrations (MFC) [31]. Yeast cultures were adjusted to 1.0 × 10^5^ CFU/per well with sterile PBS (Phosphate buffered saline, pH 7.4). The 96-well microtiter plates with serially diluted flavonoids (concentration range 0.3–75 µg/mL) in liquid broth were incubated at 37 °C for 24 h. After incubation, MIC and MFC were determined. The lowest concentrations that were not inducing microscopically observed growth were considered as MIC. For microscopic determination of growth, we used inverted microscope Nikon Eclipse TS2 (Amsterdam, Netherland) and examined the fungal growth in the wells of 96-well microtiter plates compared to the control (untreated yeast cells). MFC values were determined as concentrations without visible growth after serial sub-cultivation of 10 µL of samples at 37 °C for 24 h. Ketoconazole and amphotericin B (SigmaAldrich, Darmstadt, Germany) were used as an antifungal control. The tested flavonoids were obtained from Extrasynthese, Rhone, France.

### 4.3. Impact of Selected Compounds on Candida albicans Virulence Factors

#### 4.3.1. Antibiofilm Activity

Potential of selected compounds to interfere with *C. albicans* 475/15 biofilm formation was investigated as previously described [39]. Briefly, *C. albicans* 475/15 was incubated with serial dilution of the compounds (MIC and subMICs) in YPD medium, in 96-well microtiter plates with adhesive bottom (Sarstedt, Nümbrecht, Germany), at 37 °C for 24 h. After incubation, wells were washed twice with sterile PBS and methanol was added into each well. After fixation, methanol was discarded, and the plate was air dried. Formed biofilms were stained with 0.1% crystal violet (Bio-Merieux, Craponne, France) for 30 min. The plate was gently washed under the tap of water and air dried. The stain bound to the remaining biofilms was dissolved with ethanol (96%, Zorka, Šabac, Serbia). Absorbance was read on a Multiskan™ FC Microplate Photometer, Thermo Scientific™. The percentage of inhibition of biofilm formation was calculated by the formula:

[(A620control − A620sample)/A620control] × 100

#### 4.3.2. Anti-Hyphal Forming Activity

*C. albicans* 475/15 cells were incubated with MIC of tested compounds in YPD + 10% FBS, at 37 °C, for 4 h. Cells were examined under microscope (Nikon Eclipse TS2, Amsterdam, Netherland) and number of cells, growing in the yeast or hyphal and germ tube formations, was determined. Assay was performed in triplicate and percentage of hyphae was determined.

### 4.4. Interference with Expression of CDR1 and ERG11

#### 4.4.1. RNA Isolation

Total RNA was isolated as described by Sanglard et al. [40] with some modifications. *C. albicans* 475/15 was grown with agitation at 30 °C in YEPD until OD540 = 0.4. *C. albicans* cells were incubated with MIC of compounds in YEPD at 30 °C with agitation for 30 min. Cells were centrifuged (4600 rpm, 4 °C for 5 min (Rotanata 460 R, Hettich, Tuttlingen, Germany)) and the pellet was resuspended in 300 µL of RNA buffer (0.1 M Tris-HCl pH 7.5, 0.1M LiCl, 10mM EDTA, 0.5% SDS). The pellet was homogenized with 200 µL of DEPC treated glass beads and 300 µL of Phenol:Chloroform:Isoamyl Alcohol (Sigma-Aldrich, Darmstadt, Germany) in Precellys Evolution tissue homogenizer (Bertion instruments, Montigny-le-Bretonneux, France) at 4500 rpm for 5 s. After centrifugation (14,000 rpm, 4 °C, 1 min (Universal 32 R, Hettich, Tuttlingen, Germany)) 200 µL of supernatant was vortexed with 250 µL of Phenol:Chloroform:Isoamyl Alcohol for 10 s. Samples were centrifuged (14,000 rpm, 4 °C, 1 min) and 200 µL of supernatant was transferred to new tubes, 400 µL of ice-cold 99% ethanol was added to each tube, and they were kept on dry ice for 10 min. Samples were centrifuged (14,000 rpm, 4 °C, 2 min), pellet was washed with ethanol (70%), left to dry at room temperature, after which it was dissolved in 50 µL of DEPC treated water. Nanodrop (ND-1000, Witec AG, Sursee, Switzerland) was used for determination of concentration and purity of samples. RNA was stored at −80°C.

#### 4.4.2. DNAse Treatment and cDNA Synthesis

Amount of 10 µg RNA was incubated with 5 µL of DNAse buffer and 1 µL of DNAse at 37 °C for 30 min (DNA-free™ DNA Removal Kit, Ambion, Bleiswijk, Netherlands). After addition of DNAse inactivation reagent tubes were kept at room temperature with occasional vortexing for 5 min. Samples were centrifuged (14,000 rpm, 4°C, 2 min) and the concentration and quality of supernatant were analyzed on Nanodrop (ND-1000, Witec AG, Sursee, Switzerland).

Synthesis of cDNA was done with a Transcriptor High Fidelity cDNA synthesis kit (Roche). RNA (1 µg) was incubated with 60 µM random hexamer primers, in 11.4 µL of total sample volume, at 65 °C for 10 min. The mixture of Transcriptase reaction buffer 5×, Deoxynucleotide mix, DTT, Protector RNAse inhibitor, and Transcriptor High Fidelity Reverse Transcriptase was added to each tube according to manufacturer instructions. Synthesis of cDNA was completed by incubating the mixture at 50 °C for 30 min and 85 °C for 5 min (Peqstar thermocycler, Peqlab, Erlangen, Germany).

#### 4.4.3. qPCR

Wells of MicroAmp Fast 96-Well Reaction plate ((0.1 mL), Applied biosystems, Waltham, MA, USA) were incubated with 10 µL of mixture (cDNA, 2× MasterMix, primers, probe (Table 3), and water) [41]. Analysis was performed in StepOnePlus Real Time PCR system with relative standard curve method. Expression levels of *CDR1* and *ERG11* were normalized to expression of *ACT1*.

### 4.5. Cytotoxicity of Compounds Towards Porcine Liver Primary Cells

Preparation of PLP2 cell line was performed with freshly harvested porcine liver supplied from a local slaughter house. Tissues from the liver were washed in Hank’s balanced salt solution comprising 100 U/mL penicillin and 100 μg/mL streptomycin and split into 1 × 1 mm^3^ explants. Some of the prepared explants were set in 25 cm^2^ tissue flasks in Dulbecco’s modified Eagle’s medium (DMEM) with 10% fetal bovine serum (FBS), 2 mM nonessential amino acids, 100 U/mL penicillin, and 100 mg/mL streptomycin, and incubation was conducted at 37 °C with 5% CO_2_ in a humidified atmosphere. The fresh medium was added every 2 days. Cultivation of the cells was further conducted with direct observation by the phase contrast microscope every 2–3 days. Before confluence was achieved, cells were subcultured and seeded in 96-well plates at a density of 1.0 × 10^4^ cells per well and cultivated in DMEM supplemented with 10% FBS, 100 U/mL penicillin, and 100 μg/mL streptomycin. Previously described Sulforhodamine B assay [42] was used for the determination of cytotoxicity. The obtained results were defined as GI_50_ values corresponding to the compound concentration that inhibits 50% of the net cell growth. Ellipticine was used as a positive control.

### 4.6. Statistical Analysis

The antifungal results are expressed as mean values of three replicates and standard deviation (SD). The results were analyzed using one-way analysis of variance (ANOVA) followed by Tukey’s HSD test with *α* = 0.05 using the SPSS v. 18.0 program. QPCR analysis was done in technical triplicates with the results presented as mean values of 2 biological replicates.

## 5. Conclusions

The lowest capacity against planktonic fungal cells indicated by the highest value of minimal inhibitory concentration was recorded for quercetin. Overall, the examined flavonoids have shown promising antifungal activity. The tested molecules, especially apigetrin and isoquercitrin, have notably lowered potential of *Candida albicans* to establish biofilm, with its antivirulence potential additionally emphasized by their anti-hyphal properties elucidating the potential of these natural products to be developed as antivirulence therapeutics. Another mode of antifungal therapy could also be based on lowering expression of resistance linked genes by providing elimination of candidiasis with lower concentrations of current therapeutics. In this manner, the activity of apigenin and apigetrin (lowering *CDR1* expression) and apigenin and rutin (lowering *ERG11* expression) is of great importance. On the other hand, undesirable activity was recorded for isoquercitrin (increased *ERG11* expression). All of the studied flavonoids, with the exception of quercitrin, were able to exhibit in vitro fungicidal effects in concentrations that were not cytotoxic indicating them as promising antifungal candidates.

The obtained results confirm great biological potential among the molecules from flavonoid class—this time in the terms of novel antifungal development. In this perspective, future studies might further enlighten their antifungal capacities especially by the in vivo experiments on different candidiasis models.

## Figures and Tables

**Table 1:** Antifungal activity of tested flavonoids, values are means ± SD in µg/mL of three replicates. Different letters (a, b, c, d) in each column indicate a significant statistical difference between the samples (*p* < 0.05). Minimum inhibitory concentration (MIC) and minimum fungicidal concentration (MFC) values of the compounds are compared separately for each of the fungal strain tested.

Compounds	*C. albicans* 475/15		*C. albicans* 527/14		*C. albicans* 10/15		*C. albicans* 13/15	
	MIC	MFC	MIC	MFC	MIC	MFC	MIC	MFC
Luteolin	37.5 ± 1 ^c^	75 ± 2 ^c^	37.5 ± 1 ^c^	75 ± 20 ^c^	37.5 ± 1 ^c^	75 ± 2 ^c^	37.5 ± 1 ^c^	75 ± 2 ^c^
Quercetin	75 ± 2 ^d^	150 ± 10 ^d^	75 ± 2 ^d^	150 ± 10 ^d^	75 ± 3 ^d^	150 ± 20 ^d^	75 ± 10 ^d^	150 ± 20 ^d^
Quercitrin	37.5 ± 2 ^c^	75 ± 3 ^c^	37.5 ± 2 ^c^	75 ± 3 ^c^	37.5 ± 2 ^c^	75 ± 3 ^c^	37.5 ± 1 ^c^	75 ± 3 ^c^
Isoquercitrin	37.5 ± 1 ^c^	75 ± 2 ^c^	37.5 ± 1 ^c^	75 ± 2 ^c^	37.5 ± 2 ^c^	75 ± 2 ^c^	37.5 ± 2 ^c^	75 ± 2 ^c^
Rutin	37.5 ± 1 ^c^	75 ± 1 ^c^	37.5 ± 1 ^c^	75 ± 1 ^c^	37.5 ± 1 ^c^	75 ± 2 ^c^	37.5 ± 1 ^c^	75 ± 2 ^c^
Ketoconazole	3.1 ± 0.1 ^b^	6.2 ± 0.1 ^b^	3.1 ± 0.1 ^b^	6.2 ± 0.1 ^b^	3.1 ± 0.1 ^b^	50 ± 0.1 ^b^	1.6 ± 0.2 ^b^	50 ± 0.2 ^b^
Amphotericin B	0.63 ± 0.001 ^a^	1.25 ± 0.002 ^a^	0.63 ± 0.001 ^a^	1.25 ± 0.002 ^a^	0.63 ± 0.001 ^a^	1.25 ± 0.002 ^a^	0.63 ± 0.001 ^a^	1.25 ± 0.002 ^a^

MIC—minimal inhibitory concentration, MFC—minimal fungicidal concentration.

**Table 2:** Cytotoxicity of tested compounds, GI_50_ (compound concentration that inhibited 50 % of the net cell growth) expressed in µg/mL.

Compound	GI_50_
Apigenin	>400
Apigetrin	90 ± 1
Luteolin	>400
Quercetin	>400
Quercitrin	73 ± 3
Isoquercitrin	>400
Rutin	>400
Ellipticin	3.22 ± 0.2

**Table 3:** Sequences of TaqMan primers and probes used in qPCR.

Primer	Sequence
CDR1-ORF-F	ATGACTCGAGATATTTTGATA
CDR1-ORF-R	TTAACAGCAATGGTCTTTA
ERG11-ORF-F	ATTGTTGAAACTGTCATTG
ERG11-ORF-R	CCCCTAATAATATACTGATCTG
ACT-ORF-F	GCATCACACTTTTTACAAT
ACT-ORF-R	AAACATAATTTGAGTCATCTTT
**Probe**	**Sequence**
CDR1-P2	CATTATGAGACCTGGTGAACTTACT
ERG11-P2	TTTGTCCCTTAGTGTTACACA
ACT1-P2	TTGCTCCAGAAGAACATCCAGT

## Data Availability

The data presented in this study are available in this article.
